# Insecticide-treated bed net access and use among preschool children in Nouna District, Burkina Faso

**DOI:** 10.1093/inthealth/ihaa003

**Published:** 2020-02-29

**Authors:** Ali Sié, Mamadou Bountogo, Mamadou Ouattara, Pascal Zabre, Cheik Bagagnan, Alphonse Zakane, Jessica Brogdon, Elodie Lebas, Ying Lin, William W Godwin, Till Bärnighausen, Thomas M Lietman, Catherine E Oldenburg, the Étude CHAT Study Group

**Affiliations:** 1 Centre de Recherche en Santé de Nouna, Rue Namory Kéita, Nouna, Burkina Faso; 2 Francis I. Proctor Foundation, University of California, 513 Parnassus Ave, San Francisco, San Francisco, CA, USA, 94143; 3 Heidelberg Institute of Global Health, University of Heidelberg, Im Neuenheimer Feld 130/3, 69120 Heidelberg, Germany; 4 Africa Health Research Institute, Somkhele, South Africa; 5 Department of Global Health and Population, Harvard T.H. Chan School of Public Health, Boston, MA, USA; 6 Department of Ophthalmology, University of California, San Francisco, San Francisco, CA, USA; 7 Department of Epidemiology & Biostatistics, University of California, San Francisco, San Francisco, CA, USA

**Keywords:** Burkina Faso, insecticide-treated bed nets, malaria

## Abstract

**Background:**

We evaluated universal insecticide-treated bed net access and use in children <5 y of age in a rural area of Burkina Faso.

**Methods:**

A door-to-door enumerative census was conducted in Nouna District, Burkina Faso in December 2018 through April 2019. The most recent mass bed net distribution campaign occurred in June 2016. Heads of households were interviewed about household bed net ownership and use by children <5 y of age. We evaluated the relationship between demographic and socio-economic factors and household universal bed net access and use by children.

**Results:**

In 23 610 households with at least one child <5 y of age, 71 329 bed nets were reported (94.5% insecticide-treated). One-third (35.2%) of households had universal access and two-thirds (67.0%) of children slept under an insecticide-treated net the previous night. Children in households with universal access more often slept under a net the previous night (adjusted odds ratio 4.81 [95% confidence interval 4.39–5.26]).

**Conclusions:**

Bed net coverage was substantially less than the 80% World Health Organization target for universal coverage in Nouna District. Insecticide-treated nets were used preferentially for children, but important gaps remain in consistent bed net use in this population. Structural and behavioural interventions are needed to close these gaps.

## Introduction

Long-lasting insecticidal nets (LLINs) have been shown to reduce child mortality and *Plasmodium falciparum* infection compared with no bed net use or use of untreated nets.^1^ The World Health Organization (WHO) recommends that mass bed net distribution campaigns distribute one net for every two persons in a household, with distribution repeated every 3 y and continuous distribution via antenatal care and the expanded programme on immunization (EPI).^2^ The first national insecticide-treated bed net campaign in Burkina Faso took place in 2010.^3^^,^^4^ This campaign led to nearly all households reporting owning at least one bed net and >50% of households reporting that they owned at least one net for every two people.^4^

In regions with seasonal malaria transmission, such as Burkina Faso, the use of LLINs can vary dramatically, with use decreasing in the dry months when mosquitoes are less active.^3^ Despite overall lower risk of infection, a large percentage of children may still be affected by symptomatic or asymptomatic malaria even in the low transmission season.^5–7^ Surveys have indicated that the prevalence of children sleeping under a bed net has increased markedly in Burkina Faso over time.^8^ However, there may still be gaps in coverage particularly during the low transmission season or several years after mass bed net distribution campaigns, which may place children at risk of malaria infection and adverse outcomes. Here we report the prevalence of universal coverage of LLINs in households with children <5 y of age and children’s use of LLINs in a region of Burkina Faso with seasonal transmission of malaria.

## Materials and methods

### Study design

From December 2018 to April 2019, a door-to-door enumerative census was undertaken in 228 rural villages in 10 communes of Nouna District, Burkina Faso that are not part of the Nouna Health and Demographic Surveillance System (HDSS).^9^ Trained census workers went door-to-door in each village in the study area and visited each structure in the village. Structures were classified as inhabited or uninhabited and Global Positioning System coordinates were obtained for all structures. In inhabited structures, the head of each household was interviewed. The head of each household listed the number of male and female residents in their household, answered questions related to the household’s structure and assets, and reported information for each resident member of the household. For each member of the household, information was collected related to the individual’s date of birth and how the birthdate was verified (e.g. via birth certificate, health card, passport, etc.) and their sex, religion, ethnicity, education, marital status and occupation. For children <5 y of age, the caregiver was asked if the child’s mother resided in the home. The study was reviewed and approved by the institutional review boards at the Comité National d’Ethique pour la Recherche (National Ethics Committee of Burkina Faso; protocol 2018-8-11) and the University of California, San Francisco (protocol 17-24230). Written informed consent was obtained from the head of each household for their household to participate in the census.

### Study setting

The study area covers approximately two-thirds of Nouna District in northwestern Burkina Faso, extending to the Mali border. The area is rural agrarian, where most inhabitants are subsistence farmers and cattle keepers. The climate features highly seasonal rainfall, with the rainy season lasting approximately from July to October, which coincides with the high malaria transmission season. Monthly seasonal malaria chemoprevention (SMC) is distributed to children 3–59 months of age during this period (four distributions). No SMC distributions occurred during the period of data collection. The most recent mass bed net distribution campaign in the study area was in June 2016, and bed nets are distributed during antenatal care.

### Bed net measures

Heads of households were asked if the household owned bed nets (‘Does the household have mosquito nets?’) and, if so, if they owned insecticide-treated bed nets (‘What kind?’, with options including impregnated, not impregnated or do not know). They were then asked how many nets the household owned. Universal bed net access was defined as the household possessing at least one bed net for every two residents, which was calculated by dividing the number of bed nets by the number of residents in the household as measured in the census. The head of household was asked if each child in the household slept under a bed net the night before. Heads of households were asked to report if the child slept under a bed net and, if so, if it was an impregnated, non-impregnated or unknown type of bed net.

### Covariates

We investigated factors associated with insecticide-treated bed net access and use at the household and individual level. At the household level, the number of residents in each household was calculated based on the census. The type of toilet used most commonly by the household was categorized as improved latrines (e.g. with a slab), latrines without a slab or no latrine or toilet. A principal components analysis was used to calculate a household resources score based on the head of household report about ownership of 20 different items, including radios, televisions, refrigerators, stoves, telephones, bicycles, motorcycles and lamps. Finally, we included the number of rooms in the house as reported by the head of the household. At the individual level, we included child’s sex, age in years, ethnicity, religion and if the child’s mother was in residence in the house.

### Statistical analysis

Descriptive characteristics were calculated overall and by household universal access to bed nets using medians and interquartile ranges (IQRs) for continuous variables and proportions for categorical variables. We first modelled household-level characteristics associated with universal insecticide-treated bed net access at the household level using bivariate and multivariable logistic regression models, with household-level independent variables including the number of residents in the household, the household’s primary toilet type, the results of the principal component analysis of the household’s resources and the number of rooms in the household. This analysis was done at the household level and thus was not adjusted for clustering. We then modelled factors associated with children sleeping under an insecticide-treated bed net the previous night using bivariate and multivariable logistic regression models with standard errors adjusted for clustering at the household level. Independent variables in these models included the household-level factors previously described as well as if the household had universal insecticide bed net access and individual-level factors including the child’s sex, age in years, ethnicity, religion and if their mother resided in the house. All tests were two-sided and p<0.05 was considered statistically significant. All analyses were performed using Stata 15.1 (StataCorp, College Station, TX, USA).

**Table 1:** Household and child characteristics by universal bed net access in the household

Characteristics	Universal bed net access	No universal bed net access	Overall
Households, N	8317	15 293	23 610
Residents, n, median (IQR)	6 (4–8)	8 (6–11)	7 (5–10)
Primary toilet type, n (%)			
Improved latrine	570 (6.9)	815 (5.3)	1385 (5.9)
Unimproved latrine	4262 (51.2)	7614 (49.8)	11 876 (50.3)
None	3485 (41.9)	6864 (44.9)	10 349 (43.8)
Household resources^a^, median (IQR)	−0.12 (−1.03–1.13)	−0.18 (−1.07–0.70)	−0.12 (−1.03–0.98)
Rooms in the house, n, median (IQR)	3 (2–4)	3 (2–4)	3 (2–4)
Children, N	13 834	32 514	46 348
Female, n (%)	6749 (48.8)	15 790 (48.6)	22 539 (48.6)
Age (months), median (IQR)	29 (14–44)	30 (15–45)	30 (15–45)
Ethnicity, n (%)			
Dafing/Marka	3995 (28.9)	7311 (22.5)	11 306 (24.4)
Bwaba	4444 (32.1)	9136 (28.1)	13 580 (29.3)
Mossi	970 (7.0)	2444 (7.5)	3414 (7.4)
Samo	600 (4.3)	1145 (3.5)	1745 (3.8)
Peulh	1631 (11.8)	5877 (18.1)	7508 (16.2)
Other	2195 (15.9)	6601 (20.3)	8795 (19.0)
Religion, n (%)			
Muslim	8739 (63.2)	22 581 (69.5)	31 320 (67.6)
Catholic	3578 (25.9)	6205 (19.1)	9783 (21.1)
Protestant	1092 (7.9)	2354 (7.2)	3446 (7.4)
Animist	385 (2.8)	1262 (3.4)	1647 (3.6)
Other	40 (0.3)	112 (0.3)	152 (0.3)
Child’s mother resides in the house, n (%)	13 135 (95.0)	31 026 (95.4)	44 161 (95.3)
Child slept under bed net last night, n (%)	12 018 (86.7)	19 027 (58.5)	31 045 (67.0)

**Table 2:** Household factors associated with universal bed net access in the household (N=23 610)

Factors	Bivariate		Multivariable	
	OR (95% CI)	p-Value	aOR (95% CI)	p-Value
Number of residents	0.87 (0.86–0.88)	<0.001	0.78 (0.77–0.79)	<0.001
Primary toilet type				
Improved latrine	1.00		1.00	
Unimproved latrine	0.80 (0.71–0.90)	<0.001	1.09 (0.95–1.24)	0.21
None	0.73 (0.65–0.81)	<0.001	0.99 (0.87–1.13)	0.89
Household resources	1.07 (1.05–1.09)	<0.001	1.17 (1.14–1.19)	<0.001
Number of rooms in the house	0.98 (0.97–1.00)	0.005	1.29 (1.27–1.32)	<0.001

## Results

A total of 36 065 households were identified, of which 23 610 (65.5%) had at least one child <5 y of age in residence at the time of the census and data on bed net use. Data on bed net use were missing from four households with children <5 y of age in residence. In households with children <5 y of age, a total of 71 329 bed nets were reported, of which 67 431 (94.5%) were insecticide treated, for 193 760 residents. This translated to 8317 (35.2%) households being classified as having at least one insecticide-treated bed net for every two residents. Caregivers of 46 348 children were interviewed, of whom 31 045 (67.0%) slept under an insecticide-treated net the previous night. The median age of children in this study was 30 months (IQR 15–45) and 48.6% were female (Table 1). The mother of most children (95.3%) was resident in the house.

In a multivariable model, households with a larger number of residents had reduced odds of universal access to insecticide-treated bed nets (adjusted odd ratio [aOR] 0.78 [95% confidence interval {CI} 0.77–0.79], p<0.001). Wealthier households, as indicated by a greater number of resources in the households and more rooms in the house, had increased odds of universal access to an insecticide-treated net (Table 2). There was no difference in bed net access by type of latrine used by the household.

**Table 3:** Multilevel factors associated with sleeping under a bed net the previous night among children <5 y of age (N=46 348)

Factors	Slept under bed	Bivariate	Multivariable
	net, n (%)	OR (95% CI)	p-Value	aOR (95% CI)	p-Value
		Household-level factors
Number of residents in household	n/a	1.00 (0.99–1.00)	0.28	1.01 (1.00–1.02)	0.08
Primary toilet type	n/a				
Improved latrine		1.00		1.00	
Unimproved latrine		0.69 (0.60–0.79)	<0.001	0.76 (0.65–0.89)	0.001
None		0.71 (0.62–0.82)	<0.001	0.91 (0.78–1.07)	0.24
Household resources	n/a	1.13 (1.11–1.16)	<0.001	1.12 (1.09–1.15)	<0.001
Number of rooms in the house	n/a	1.01 (0.97–1.05)	0.68	1.00 (0.99–1.00)	0.39
Household has universal bed net access	n/a	4.69 (4.33–5.08)	<0.001	4.81 (4.39–5.26)	<0.001
		Child-level factors			
Child’s sex					
Male	67.2%	1.00		1.00	
Female	66.7%	0.98 (0.94–1.02)	0.25	0.95 (0.91–1.00)	0.04
Age (years)					
0	71.5%	1.00		1.00	
1	69.0%	0.89 (0.83–0.95)	<0.001	0.89 (0.82–0.95)	0.001
2	67.0%	0.81 (0.76–0.86)	<0.001	0.82 (0.77–0.88)	<0.001
3	65.0%	0.74 (0.70–0.78)	<0.001	0.76 (0.71–0.81)	<0.001
4	62.9%	0.68 (0.64–0.72)	<0.001	0.71 (0.66–0.76)	<0.001
Ethnicity					
Dafing/Marka	72.3%	1.00		1.00	
Bwaba	64.3%	0.69 (0.64–0.75)	<0.001	0.60 (0.52–0.69)	<0.001
Mossi	68.0%	0.81 (0.71–0.92)	0.001	0.78 (0.68–0.90)	0.001
Samo	75.0%	1.14 (0.94–1.39)	0.18	1.16 (0.92–1.46)	0.20
Peulh	64.9%	0.71 (0.64–0.78)	<0.001	0.82 (0.73–0.92)	0.001
Other	64.0%	0.68 (0.61–0.76)	<0.001	0.72 (0.64–0.81)	<0.001
Religion					
Muslim	67.5%	1.00		1.00	
Catholic	67.7%	1.00 (0.93–1.07)	0.98	1.23 (1.09–1.41)	0.001
Protestant	65.4%	0.91 (0.82–1.01)	0.07	1.21 (1.03–1.42)	0.02
Animist	56.0%	0.61 (0.53–0.71)	<0.001	0.83 (0.68–1.01)	0.06
Other	71.1%	1.18 (0.73–1.88)	0.49	1.81 (1.05–3.11)	0.03
Child’s mother resides in the house					
No	60.8%	1.00		1.00	
Yes	67.3%	1.33 (1.20–1.46)	<0.001	1.31 (1.17–1.46)	<0.001

^a^Numbers not provided for household-level characteristics due–continuous nature of the variable and because sleeping under a bed net is an individual-level characteristic.

In a multilevel logistic regression model, children who lived in households with universal access had increased odds of sleeping under an insecticide-treated bed net the previous night (aOR 4.81 [95% CI 4.39–5.26], p<0.001). Older children had reduced odds of sleeping under an insecticide-treated bed net compared with younger children (Table 3; p for trend <0.001). Children whose mothers lived in the same house had increased odds of sleeping under a net the previous night (aOR 1.31 [95% CI 1.17–1.46], p<0.001). There was evidence of a significant difference in bed net use by ethnicity (Table 3; overall p<0.001) and religion (Table 3; p<0.001) of the child. Similar to results for universal access, children in households with greater resources had increased odds of sleeping under a bed net (Table 3). There was no evidence of a difference in bed net use by size of the household in terms of the number of residents or number of rooms.

## Discussion

Approximately one-third of households in communities of Nouna District outside of the Nouna HDSS area met the criteria for universal access to an insecticide-treated net (at least one bed net per two residents),^10^ far below the WHO target of 80% access.^11^ Approximately two-thirds of children <5 y of age slept under an insecticide-treated net the previous night, suggesting that families without universal access prioritize bed net coverage for their young children. However, children living in households with universal access had markedly increased odds of sleeping under an insecticide-treated net compared with those who did not, underscoring the importance of ensuring bed net access to all households. There were differences in the odds of sleeping under a bed net across demographic characteristics, including ethnicity and religion, suggesting that the use of bed nets is not homogeneous in this population. Interventions addressing disparities in bed net use may need to consider demographic differences in access and the use of bed nets.

Mass bed net distribution campaigns have been successful at increasing ownership of insecticide-treated nets in rural Burkina Faso.^4^ While these campaigns have increased the number of households owning at least one bed net, they have not ubiquitously resulted in universal coverage. In Nouna District, the mass bed net distribution campaign resulted in approximately 50% coverage of universal access.^4^ In the present study, universal access was lower approximately 2.5 y after the most recent mass bed net distribution than previously reported after the first bed net distribution in 2011. This may be due to the growth of families since the last campaign or destruction of older nets. Previous studies in other settings showed that universal coverage dropped to baseline levels 3 y after a universal bed net campaign.^12^ Similar to previous studies,^4^ larger families had decreased odds of having universal bed net access, probably due to the fact that more nets are required for larger families. Household socio-economic status was independently associated with increased bed net access, suggesting that there may be disparities in equity of universal access.

At the individual level, younger children more often slept under a bed net the previous night compared with older children. While younger age does not necessarily correlate with increased risk of asymptomatic malaria among children <5 y of age,^13^^,^^14^ younger children may be more likely to have severe consequences from malaria infection.^15^ Parents may preferentially use bed nets for younger children or these households may be more likely to have a functional bed net, as bed nets are routinely distributed as part of antenatal care. Expanding universal access may help ensure that older children and other household members receive protection from bed nets.

This analysis must be considered in the context of some limitations. All bed net measures were measured as reported by the head of the household during the census, and we did not objectively confirm the head of household report (e.g. by counting the number of bed nets inside a structure). As the benefit of bed net use is generally common knowledge, heads of households may have over-reported bed net use, which could lead to an overestimate of bed net use in this population. Heads of households may have been more likely to misreport a child’s use of a bed net than the child’s direct caregiver, which could introduce misclassification. The type of bed net used (insecticide-treated or untreated) was not objectively verified and the use of insecticide-treated nets may have been over-reported or misclassified by respondents. We did not collect data on malaria parasitaemia in Nouna District at the time this census was conducted. This census was conducted during the low malaria transmission season and, most probably, bed net use was lower than it would have been during the high transmission season. However, we cannot comment on the burden of malaria in the study communities at the time of data collection. The timing of data collection in this study was based on timing for preparation for a large-scale community randomized trial of azithromycin distribution for prevention of child mortality.^16^ The study was therefore not designed specifically to evaluate bed net use in a given season, but data collection happened to occur during the low malaria transmission season. This study is likely to be limited in its generalizability to different seasons and geographies. Patterns of bed net use may differ by time and areas with different malaria transmission patterns or in areas with differences in factors that are related to bed net use.

## Conclusions

Here we demonstrate that universal bed net access was suboptimal in a large portion of Nouna District, Burkina Faso approximately 2.5 y after the last mass bed net distribution. Although the study was conducted during the low transmission season, this could lead to increased vulnerability to malaria during the high transmission season in the absence of a new bed net campaign or alternative distribution mechanism. Although children <5 y of age appeared to preferentially have access to bed nets, as the prevalence of bed net use in this age group was higher than the prevalence of universal access, there were still gaps in bed net coverage in this age group. Ensuring that all household members have access to an insecticide-treated net is an important component of a comprehensive malaria prevention strategy. It is likely that concerted strategies of structural (e.g. mass distribution campaigns) and behavioural (e.g. communication to improve utilization of existing nets^17^) interventions are needed to close the important gaps in bed net coverage and use.
